# Removal of an Aural Foreign Body by Magnetism

**DOI:** 10.5811/cpcem.24845

**Published:** 2025-01-01

**Authors:** Emily Prentice, Emily Bartlett, Jonathan S. Ilgen

**Affiliations:** * University of Washington School of Medicine, Seattle, Washington; † University of New Mexico, Albuquerque, Department of Emergency Medicine, New Mexico; ‡ University of Washington, Department of Emergency Medicine, Seattle, Washington

**Keywords:** aural foreign body, magnetic bead

## Abstract

**Case Presentation:**

A male patient in his thirties with a history of polysubstance use presented to the emergency department (ED) due to an abrasion on his left forehead caused by banging his head against a wall in self-injurious behavior. A non-contrast computed tomography of the head obtained to rule out intracranial injury incidentally demonstrated a radiodense foreign body in the left external ear canal. A round metallic foreign body was subsequently visualized on otoscopic examination. The aural foreign body (AFB) was identified as a metallic bead that the patient had placed into his own ear; however, he reported no associated discomfort, hearing changes, or discharge. Traditional approaches for removing AFBs were considered; however, due to the position and smooth surface of the bead, there was concern they would be unsuccessful. Recognizing the metallic nature of the AFB, the clinician held a ceramic donut magnet adjacent to the patient’s ear and subsequently extracted the AFB without complication or patient discomfort.

**Discussion:**

Aural foreign bodies account for a significant number of visits to EDs annually. Removal of AFBs can be challenging, often requiring specialized equipment or specialty referral for management. Using magnetism over short distances for the purpose of extracting metallic AFBs presents a low-cost, low-risk intervention. When used in applicable scenarios, this technique can decrease the need for specialty referral and can especially benefit patients seeking care in less-resourced settings.

## CASE PRESENTATION

**Image 1:**
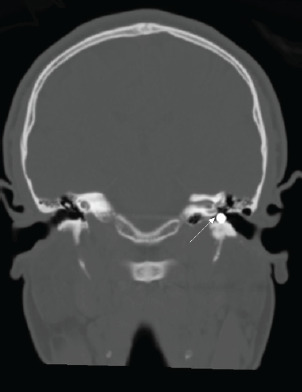
Coronal view of non-contrast computed tomography of the head. White arrow points to metallic foreign body in the patient’s left external auditory canal.

A male patient in his thirties with a history of polysubstance use was evaluated in the emergency department after hitting his head against a wall in self-injurious behavior. He had an abrasion to his left forehead and an otherwise unremarkable physical examination. A non-contrast computed tomography of the head was obtained, which ruled out intracranial injury. This imaging incidentally demonstrated a radiodense foreign body in the left external auditory canal, and a round metallic foreign body was subsequently visualized on otoscopic examination (Image 1).

When questioned, the patient reported placing a string of magnetic beads in his left ear, previously using the magnetic forces of other beads to remove these foreign bodies on his own. He was aware that a metallic foreign object remained in his external auditory canal but had been unsuccessful in attempts to remove it. He experienced no discomfort or other auditory complaints in the context of this foreign body.

**Image 2:**
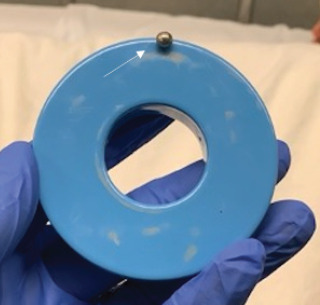
White arrow pointing to magnetic foreign body on the ceramic donut magnet after removal from the patient’s external auditory canal.

We considered several approaches to remove the aural foreign body (AFB).^1^ Due to the positioning of the round sphere in the ear canal, a speculum or jet of fluid would not have been able to reach around it and would likely have pushed the AFB further into the canal. Use of cyanoacrylate glue on the tip of a swab was also considered; however, there was concern it would not adhere well to the smooth metal surface. Specialized suction catheters were not readily available in our ED. A ceramic donut magnet from the code cart—typically used to temporarily induce pacemakers into asynchronous mode—was obtained. When the donut magnet was held adjacent to the patient’s left ear, the magnetic sphere emerged from the ear canal and adhered to the magnet (Image 2). The procedure was well tolerated by the patient with no reported discomfort or complications.

## DISCUSSION

Spherical, non-graspable foreign bodies often require specialist referral and potential removal under anesthesia.^1^ In this case, we quickly and easily removed a magnetic spherical foreign object using a ceramic donut magnet, obviating the need for specialty consultation. Although the use of magnetized instruments for this purpose has been described anecdotally,^2^ to our knowledge this is the first published report of removal of an AFB by magnetic force acting over a distance. Magnets are commonly found in EDs, and this technique could be readily adopted without the need to purchase specialized equipment. Additionally, the use of magnets to remove AFBs may be applicable to objects that are permanent magnets as well as metallic foreign bodies susceptible to induced magnetism.^3^

**CPC-EM Capsule**

What do we already know about this clinical entity?

*Removal of aural foreign bodies (AFB) can be challenging, often requiring specialized equipment or specialty referral for management*.

What is the major impact of the image(s)?

*Using a ceramic donut magnet, a device commonly available in emergency departments, to remove a metallic AFB is a low-cost and easily accessible intervention*.

How might this improve emergency medicine practice?

*This low-cost, low-risk intervention would be especially useful in settings with less access to specialty care*.

A study using the National Electronic Injury Surveillance System reported that over a five-year period 250,000 ED visits were due to AFBs.^4^ However, access to otolaryngology for specialized management varies widely depending on location, and 65.7% of counties in the United States do not have a practicing otolaryngologist.^5^ This case describes the use of a low-cost, low-risk intervention for removal of magnetic foreign bodies, which can aid in patient care especially in lower resource settings with less access to specialty care.
